# Understanding the Attributes of Implementation Frameworks to Guide the Implementation of a Model of Community-based Integrated Health Care for Older Adults with Complex Chronic Conditions: A Metanarrative Review

**DOI:** 10.5334/ijic.2516

**Published:** 2017-06-27

**Authors:** Ann McKillop, Jay Shaw, Nicolette Sheridan, Carolyn Steele Gray, Peter Carswell, Walter P Wodchis, Martin Connolly, Jean-Louis Denis, G Ross Baker, Timothy Kenealy

**Affiliations:** 1 University of Auckland, NZ; 2 Women’s College Hospital, CA; 3 Bridgepoint Health, CA; 4 University of Toronto, CA; 5 University of Montreal, CA

**Keywords:** implementation, integrated care, frameworks, metanarrative review, models, older adults, primary health care

## Abstract

**Introduction:**

Many studies have investigated the process of healthcare implementation to understand better how to bridge gaps between recommended practice, the needs and demands of healthcare consumers, and what they actually receive. However, in the implementation of integrated community-based and integrated health care, it is still not well known which approaches work best.

**Methods:**

We conducted a systematic review and metanarrative synthesis of literature on implementation frameworks, theories and models in support of a research programme investigating CBPHC for older adults with chronic health problems.

**Results:**

Thirty-five reviews met our inclusion criteria and were appraised, summarised, and synthesised. Five metanarratives emerged 1) theoretical constructs; 2) multiple influencing factors; 3) development of new frameworks; 4) application of existing frameworks; and 5) effectiveness of interventions within frameworks/models. Four themes were generated that exposed the contradictions and synergies among the metanarratives. Person-centred care is fundamental to integrated CBPHC at all levels in the health care delivery system, yet many implementation theories and frameworks neglect this cornerstone.

**Discussion:**

The research identified perspectives central to integrated CBPHC that were missing in the literature. Context played a key role in determining success and in how consumers and their families, providers, organisations and policy-makers stay connected to implementing the best care possible.

**Conclusions:**

All phases of implementation of a new model of CBPHC call for collaborative partnerships with all stakeholders, the most important being the person receiving care in terms of what matters most to them.

## Introduction

Implementation scientists have developed a variety of frameworks intended to guide the research and practice of implementing innovations in health care [1]. A multitude of methods to promote the implementation and scale-up of innovations has fuelled a raft of literature about the use of theories, models and frameworks to comprehend methods that are more likely to effect better health outcomes, much of which has been summarized in systematic and other literature reviews [2]. The use of a theoretically informed framework to guide implementation may promote a smoother transition of change in practice that is more likely to be sustained over time [1,3,4,5]. However, translating different implementation theories, models and frameworks into actions to improve care is still challenging.

This systematic review of implementation literature using metanarrative methods has sought to identify the key dimensions and gaps in existing implementation frameworks to guide the implementation of a new model of community-based integrated care for older adults with multiple, chronic conditions.

## Background

The New Zealand and Canada research program Implementing Integrated Care for Older Adults with Complex Health Needs (iCOACH) programme involves cross-jurisdictional and interdisciplinary research to identify, evaluate and implement innovative, community-based models for chronic disease prevention and management. We seek to optimise health and health equity outcomes among individuals with multiple chronic morbidities requiring complex care through critical insights into the implementation of innovative models for the delivery of integrated community based primary health care (CBPHC). Having characterised the key dimensions of effective implementation of such models, we establish some key guiding principles to inform a framework for the systematic implementation of innovative models of CBPHC in the third phase of iCOACH.

Navigating the gaps between recommended practice, what healthcare consumers want and need and what they actually receive is fraught with complexity [2,6,7]. Attentive recognition is required of how health problems are experienced by people over time and in different contexts, and how they may be supported to have the best outcomes possible [7]. The importance of a person-centred (non-disease) focus is foremost in our consideration of how a framework may be used to guide implementation of innovative integrated CBPHC models. We intend to pay attention to how complex interventions interact with implementation factors in the determination of outcomes.

## Aim and Scope

In this metanarrative review, we sought to understand the attributes of relevant implementation frameworks as a first step to developing a framework to guide the implementation of a model of community-based integrated care for older adults with multiple, chronic conditions. Therefore, our overall review question is: What are the key dimensions and gaps in existing frameworks relevant to the implementation of person-focused, community-based integrated primary health care for older adults with multi-morbidities?

## Methods

We chose metanarrative methods as most suited to clarifying our topic given the heterogeneous nature of the topic and to highlight what different research approaches have contributed to what is currently known on this topic [8]. We were informed by three works on the method [8,9,10]. This relatively new type of review is a good fit for our inquiry because its multi-disciplinary approach using historical and philosophical perspectives helps to make sense of diverse qualitative and quantitative literature of, in contrast to more traditional systematic reviews from a single paradigm or research tradition. For example, systematic reviews of randomised controlled trials are very useful for evaluating the effectiveness of specific interventions but not for drawing together what is known from multiple disciplines and perspectives to inform complex programmes, such as our need for a comprehensive guide to implementation of an integrated and comprehensive model of care for a population with complex health needs. Metanarrative review methods enable the identification and analysis of similarities and contrasts in different traditions and disciplines, and in turn the development of higher order insights and conclusions about what is known and not known currently, in this case about implementation frameworks [8,11].

**Figure 1:**
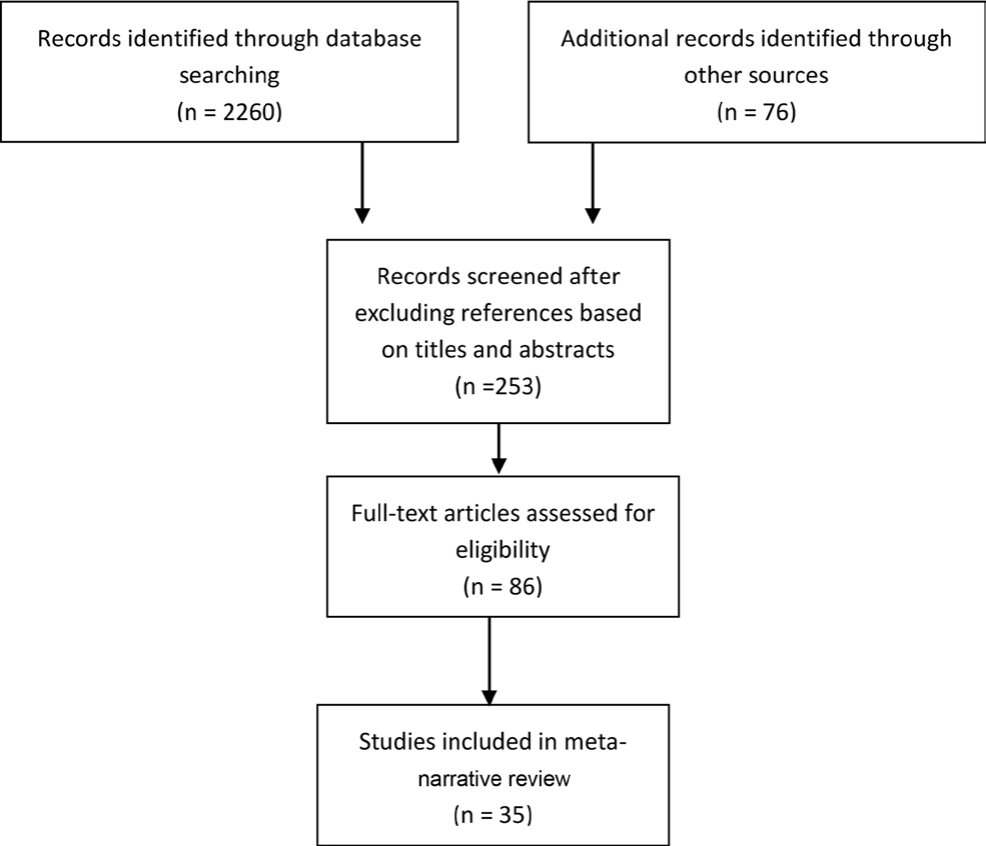
Search and selection strategy.

A preliminary, exploratory search of databases, using the terms “implementation”, “framework” and “review” in EBSCOhost (2719 hits) and Medline (7480 hits), returned many publications with inconsistent indexing of topics and irrelevant titles. We included only systematic reviews in order to capture the large literature on implementation frameworks that have been developed. Therefore, we employed a three-phase search approach. First, we identified seminal works known to the research team. At this point we broadened our search to also include articles in which frameworks had been developed from reviews of implementation-relevant theories and models in addition to ensure that we captured literature to best inform our question about the attributes of implementation. Second, we used snowballing techniques of pursuing backwards to references cited in publications as they were identified, and tracking forward the citations of seminal publications, a method said to be more efficient than hand- or database-searching (Greenhalgh 2004). Third, we employed the help of a specialist librarian to search Medline, Pubmed, CINAHL, and the Cochrane Library from 2003–2016 to check that we had already captured eligible reviews in the first two approaches. We selected this time period given substantive increases in literature on the topic after 2003. We specifically searched for review articles. Backwards citation searching identified one seminal systematic review that met our inclusion criteria, that of Wensing and Grol in 1994. The search process is presented in Figure 1 below.

We included articles in English that were published reviews of literature that specifically articulated the search and analysis methods used, and were deemed relevant to our topic. We excluded unpublished literature and reviews of implementation that focussed on single disease-states to avoid disease-specific interventions in the care of patients with multiple morbidities. For that same reason we excluded reviews of particular or limited interventions, and articles that were relevant to emergency, paediatrics, or hospital-based care because these topics were not germane to community based primary care. Appraisal was conducted independently by three authors (AM, JS, and CSG) and recorded on data extraction forms, guided by the Critical Appraisal Skills Program (CASP) checklist [12] and a sample was reviewed by AM with further checking by NS and TK. Summary of the content of articles was conducted at the same time as appraisal to further assess eligibility, direct further searching and summarise content in response to the first three questions posed in Table 1 below.

**Table 1:** Further questions posed of the included literature [adapted from Greenhalgh et al [9]].


What bodies of knowledge and research traditions are relevant to the understanding of implementation of new models of primary health care? What are the key premises, theories, and methodological approaches in these research traditions? What are the main findings and conclusions of included studies, according to each research tradition? What metanarratives are represented in this literature? What are the assumptions, approaches, findings, and conclusions of these metanarratives? What higher-order insights can be achieved from these metanarratives that may advance understanding of the selection or adaptation of a framework? What are the priorities for further research?


The metanarratives were developed through review and discussion among the authors as an iterative process based on the summary and appraisal forms and the full-text of articles. We finally reached full agreement following further discussion and re-categorisations of articles.

## Results

Thirty five reviews including 2516 studies published over a period of 22 years, met our inclusion criteria. Of 86 full-text articles reviewed (selected based on titles and abstracts), we excluded 51 because the methods stated were not consistent with a review, or the framework, theories or models presented were not directly relevant to community-based primary health care. A variety of review methods as named by authors are presented in Table 2. Having considered the nature, contribution and content of included articles as a collection, and based on thematic analysis of notes made on the data extraction forms, we agreed on five metanarratives that captured the conceptualisations, perspectives and findings of included reviews.

**Table 2:** Reviews included in Each Meta-narrative.

Metanarrative	Reviews primarily associated	Year of publication	Literature sources	Review methods as stated by authors

1. Implementation should be Informed by Theoretical Constructs	Francke AL, Smit MC, Veer AJ, and Mistiaen P.	2008	12	Systematic meta-review
	Liaw ST, Lau P, Pyett P, Furler J, Burchill M, Rowley K, et al.	2011	17	Literature review
	Stirman SW, Kimberly J, Cook N, Calloway A, Castro F, and Charns M.	2012	125	Literature review
	Flottorp SA, Oxman AD, Krause J, Musila NR, Wensing M, Godycki-Cwirko M, et al.	2013	12	Systematic review
	Chaudoir SR, Dugan AG, and Barr CH.	2013	125	Systematic review
	Braithwaite J, Marks D, and Taylor N.	2014	57	Systematic review
	Davy C, Bleasel J, Liu H, Tchan M, Ponniah S, and Brown A.	2015	77	Systematic review
2. The Relationships Between Theoretical Constructs and the Ways in Which they Impact Implementation	Davies P, Walker AE, and Grimshaw JM.	2010	285	Systematic review
	Thomas A, Menon A, Boruff J, Rodriguez AM, and Ahmed S.	2010	35	Scoping review
	Wilson PM, Petticrew M, Calnan MW, and Nazareth I.	2012	33	Systematic scoping review
	Wisdom JP, Chor KHB, Hoagwood KE, and Horwitz SM.	2014	20	Review of theories and constructs
	Tabak RG, Khoong EC, Chambers DA, and Brownson RC.	2014	61	Narrative review
3. Developing New frameworks from Theories, Constructs and Key Factors	Greenhalgh, Robert, Macfarlane, Bate and Kyriakidou.	2004	495	Metanarrative review
	Damschroder LJ, Aron DC, Keith RE, Kirsh SR, Alexander JA, and Lowery JC.	2009	19	Consolidated framework
	Ward V, House A, and Hamer S.	2009	28	Narrative review
	Gagliardi AR, Brouwers MC, Palda VA, Lemieux-Charles L, and Grimshaw JM.	2011	18	Conceptual framework
	Meyers DC, Durlak JA, and Wandersman A.	2012	27	Synthesis of frameworks
	Taylor MJ, McNicholas C, Nicolay C, Darzi A, Bell D, and Reed JE.	2014	73	Systematic review
	Schell SF, Luke DA, Schooley MW, Elliott MB, Herbers SH, Mueller NB, et al.	2013	85	Comprehensive literature review
	Moullin JC, Sabater-Hernández D, Fernandez-Llimos F, and Benrimoj SI.	2015	49	Systematic review
4. Applying Existing Frameworks in Many Ways	Helfrich CD, Damschroder LJ, Hagedorn HJ, Daggett GS, Sahay A, Ritchie M, et al.	2010	24	Critical synthesis
	Gaglio B, Shoup JA, and Glasgow RE.	2013	71	Systematic review
	Field B, Booth A, Ilott I, and Gerrish K.	2014	146	Systematic review
	Kadu MK, and Stolee P.	2015	22	Systematic review
	Kirk MA, Kelley C, Yankey N, Birken SA, Abadie B, and Damschroder L.	2016	26	Systematic review
5. Evaluating Effectiveness of Interventions within Frameworks/Models	Wensing M, and Grol R.	1994	75	Literature review
	Grimshaw J, Thomas R, MacLennan G, Fraser C, Ramsay C, Vale L, et al.	2004	235	Systematic review
	Wensing M, Wollersheim H, and Grol R.	2006	36	Structured review of reviews
	HakkennesS, and Dodd K.	2008	27	Systematic review
	Prior M, Guerin M, and Grimmer-Somers K.	2006	33	Synthesis of systematic reviews
	Boaz A, Baeza J, and Fraser A.	2011	13	Overview of systematic reviews
	Scott SD, Albrecht L, O’Leary K, Ball G, Hartling L, Hofmeyer A, et al.	2012	32	Systematic review
	Baker R, Camosso-Stefinovic J, Gillies C, Shaw E, Cheater F, Flottorp S, et al.	2015	32	Systematic review
	Lau R, Stevenson F, Ong BN, Dziedzic K, Treweek S, Eldridge S, et al.	2015	91	Synthesis of systematic reviews

### Overview of Implementation Frameworks, Models and Theories

When viewing the included articles as a whole, it was clear that many of the earlier reviews regarded the implementation of innovations as a linear process characterised by ‘science-push’ or ‘demand-pull’approaches [13]. Articles about implementation from early in the evidence-based medicine agenda were likely to focus on the effectiveness of specific implementation interventions and, therefore more amenable to research in the positivist paradigm (refer to Table 3 below).

**Table 3:** Research traditions reflected in included articles [adapted from Greenhalgh et al.[9]].


*Positivist*, assumes an external and knowable reality that is objectively measured; researcher is impartial; generalizable statements the natural and social world are producible.	*Interpretivist*, assumes a socially constructed reality, informed reconstruction; researchers are co-constructors of knowledge, of understanding and interpretation of the meaning of lived experiences; researcher’s identity and values are inevitably implicated in the research process.	*Critical*, assumes an inherently unstable social order with domination of some groups by others, e.g. patients by health professionals. Aims to (in part) help dominated groups challenge their position in society.	*Recursive* (or *integrative*), assumes subject and object, micro and macro, social structure and human agency, are reciprocally related and that the purpose of research is to explore dynamics of such relationships.


Educational and ecological theories emerged later in the literature, and were more likely to include studies in the interpretivist tradition. Frameworks relying on these theories demonstrated stronger recognition of the complexity and unpredictability of health-care settings, multiple stakeholders and layers of interaction and change. Research strategies and analyses arising from these educational and ecological theories reflected broader perspectives and sociological approaches, but may be distinguished from the critical and recursive theoretical perspectives that also emerged between 2004 – 2016. Critical and recursive perspectives are more marginally presented in the implementation science literature, but are nonetheless present in the frameworks reviewed for this study.

In summary, the “phases” of different theoretical and philosophical perspectives although not strictly time-bound represent clustering of narratives across a complex body of literature. In addition, different narratives emphasize different efforts to advance implementation science, for example by acknowledging that theoretical constructs contribute strongly to implementation frameworks (meta-narrative 1 below), and that by applying existing frameworks in many ways their general applicability may be determined (meta-narrative 4 below).

In the next sections we present the results as five metanarratives, each with a story that contributes to making sense of implementation theories, frameworks and models. Table 4 below summarises the key elements represented in each metanarrative showing where there are overlaps of disciplinary and philosophical roots and conceptualisations.

**Table 4:** Overview of Key Features of Metanarratives.

Metanarrative	Disciplinary and Philosophical roots	Definition and Scope of Implementation	General Format of Review Questions	Implementation Conceptualised as…	End-users/Beneficiaries Conceptualised as	Implementation Context conceptualised as…

1. Implementation should be Informed by Theoretical Constructs	Cognitive, ecological, socio-cultural, communication, change, adult learning and improvement theories. Social diffusion theory, organizational theory. Interpretivist, critical and recursive roots.	Necessitates the exploration of theoretical constructs embedded in healthcare to uncover the multiple factors that influence professional practice.	What theory base or constructs underpin implementation and how can they be used to guide all of its phases?	Seeks an understanding of evidence use through theory-based reasoning and decisions.	Researchers, health system decision-makers, and health care providers. Scarce mention of consumers.	Is complex and is a potent influence on how a theory may operate within a particular project or programme.
2. The Relationships Between Theoretical Constructs and the Ways in Which they Impact Implementation	Social diffusion theory and organizational theory. Quality and safety. Based on positivist, interpretive, critical and recursive research.	Consists of multiple layers, intersections, and interdependent elements that create complex adaptive system that typifies healthcare.	What are the influences, measures, and outcome aspirations that may predict success (or failure)?	Captures the factors impacting on achievement, accomplishment and execution of translating research findings effectively and rapidly into policy and practice.	Researchers, health system decision-makers, and health care providers. Scarce mention of consumers.	The context, the nature of innovation/s, and the capacity to sustain are interacting dynamics of a complex and unstable phenomenon.
3. Developing New frameworks from Theories, Constructs and Key Factors	Psychology, sociology, biomedical science, public health, sociology, business and management studies. Primarily positivist roots with pragmatist and interpretivist influences.	Implementation is to be understandable and usable for a broad audience aided by simplifying complexity, specifying relationships, for use in various contexts.	What are the key factors influencing implementation processes and outcomes, and how do they relate to one another?	A collection of activities designed to alter the behaviour of health care providers, under the influence of a variety of contextual factors.	Researchers, health system decision-makers, and health care providers.	Context exists within and outside an organization and fundamentally influences implementation.
4. Applying Existing Frameworks in Many Ways	Primarily positivist approaches, some interpretivist influence.	Implementation is a process through which knowledge/evidence is disseminated and adopted into practice.	How are implementation frameworks being used and to what effect?	A process which occurs within a particular context involving barriers and facilitators.	Researchers and individuals seeking to disseminate knowledge.	Related to the type of evidence/intervention being implemented, and micro, meso and macro level factors that can support or hinder implementation.
5. Evaluating Effectiveness of Interventions within Frameworks/Models	Psychology, sociology, biomedical science, public health, sociology, business and management studies. Positivist research base.	Implementation involves what works. Efficacy and safety are paramount. Change is more likely if strategies address contextual barriers and enablers.	What are the most effective ways to improve health care practice and health outcomes?	Use of research evidence involves employing strategies to implement improvements in patient care.	Researchers, health system decision-makers, and health care providers and ultimately patients when health outcomes are improved.	Context determines important factors and directs the approaches used to select interventions.

### Metanarrative 1: Implementation should be Informed by Theoretical Constructs

A central proposition of the first metanarrative is that theoretically-grounded implementation is more likely to improve health outcomes by informing practice and research, thereby advancing the science of implementation [14,15,16,17,18]. This narrative included seven publications representing 425 empirical research studies. Contributions categorized within this metanarrative stress the importance of understanding the theoretical constructs underpinning mechanisms that facilitate or impede change. Theory use was thought to enhance the effectiveness of interventions by guiding modification of barriers and enablers of implementation [14,17,18], by informing the design of interventions and research [15,16], and directing exploration of pathways and moderators of changes [14,15,16,17,18]. This narrative emphasises that the nature and content of the constructs on which a framework is based influences the implementation process.

In this metanarrative, implementation science involves understanding evidence use through theory-based reasoning and decisions [2]. Comprehensive implementation frameworks may be based on multiple theoretical constructs, such as adult learning, communication, diffusion of innovations, change, social marketing, attitude change, social ecology, health promotion, and quality improvement [14,15,16,17,18]. The disciplinary roots of this diverse metanarrative include evidence-based practice, quality and safety, human and organisational psychology, sociology, change management, health systems and policy. Thus, a feature of this metanarrative is the multiple research traditions represented, including a positivist approach for positing causal pathways between theoretical constructs and implementation outcomes, and an interpretivist approach to exploring the constructs relevant to the processes and understandings of implementation.

Overall, this metanarrative provides considerable support for ensuring that a comprehensive guide for implementation of change into practice should be underpinned by the theoretical constructs relevant to those health care consumers most affected what is to be implemented, the staff and services involved, the environmental context, and various influences on professional practice [14,15,16,17,18]. The ways that constructs are characterised, in part, dictates where and how attention will be devoted during the implementation process. Once understood, implementation processes can be modified through theoretically-informed interventions [14,16]. For example, Thomas et al [16] describe how theories about knowledge use might frame active learning as a process mutually refined by those who produce knowledge and those who use it [15,16,17]. From this perspective, implementation is dependent on participatory relationships between researchers and health professionals rather than simple messenger-receiver interactions [16,17].

In this metanarrative, there is recognition that the context of implementation influences how various theoretical constructs may operate. For example, implementation may target a specific level (patients and their families, healthcare providers, organisations and policy) or may cut across multiple levels and phases and the use of a theoretical model can guide implementation strategies across these levels and phases [14,17]. A framework to guide implementation of health policy broadly across multiple organisations may allude to but not specify theoretical constructs that inform patient-clinician interactions, whereas broader change theory may be explicitly represented in the framework.

Perhaps as a result of the multiple theoretical concepts represented in the literature on which this meta-narrative is based, there is lack of direction about which theoretical constructs are appropriate to specific instances of implementation. First, there is a lack of clear rationale for theory selection (single vs multiple or general vs specific theories) [14,16]. Second, importance is made of theory-based change but exactly how the theoretical constructs are to be applied is unclear and unsystematic [14,17,18]. Third, the perspectives and experiences of patients and their families are not well-represented in the theoretical models that characterize this meta-narrative. In summary, a framework/guide to implementation should be informed by theoretical constructs that are appropriate to the people, context, practice and scope of what is being implemented.

### Metanarrative 2: The Relationships Between Theoretical Constructs and the Ways in Which they Impact Implementation

Literature in this second meta-narrative sought to specify the relationships between theoretical constructs, as described in metanarrative 1 above, and their actual impacts on implementation [4,19,20,21,22]. There is a very strong central focus on identifying the specific elements present in a health care context or an implementation project prior to its initiation that might predict its likely outcome. The distinction between this meta-narrative and the first meta-narrative is not as clear as with the rest of the meta-narratives identified in our review; however, in meta-narrative 2 constructs are specified that most strongly influence implementation, the ways in which their influence is achieved, and strategies to identify their effects [19,21,22,23]. This meta-narrative included five publications representing 434 empirical research studies.

This metanarrative has its origins in the quality and safety arena with a key concern that patients receive appropriate care and avoid harm as a result of their care. Braithwaite et al [4] emphasized that the way to achieve this is through implementation science capturing the factors impacting on achievement, accomplishment and execution of translating research findings effectively and rapidly into policy and practice, exemplifying the focus on identifying how theoretical constructs relate to one another and their actual impact on implementation outcomes [4]. A feature of this metanarrative is the multiple research traditions (positivist, interpretive, critical and recursive) that are represented to lend clarity to the factors that may predict successful implementation [4,19,21]; the levels at which they operate [22,23]; the types of outcomes that indicate success [19,21,22,23]; and tools to measure outcomes [23].

Within this meta-narrative authors were focused on informing the development of appropriate outcomes for implementation processes. For example, Chaudoir et al. [23] identified implementation outcomes according to adoption, fidelity, cost, penetration, and sustainability, reporting how research had measured outcomes in each of these domains. While this approach provided a useful categorisation of existing measures, some conceptual challenges persist (e.g., the authors cite the lack of criterion validity as an ongoing problem). Specific constructs found to be most strongly associated with implementation outcomes included (a) the availability of resources, (b) effective planning, (c) capacity of the setting to adjust to change, and (d) informal support among staff and leaders.

The patient or client as the recipient of care was also scarcely mentioned in this metanarrative. When implementation factors were coded to a framework of five structural levels representing structural, organizational, provider, patient, and innovation levels, just five of 62 measures included reference to patient factors such as health-relevant beliefs, motivation, and personality traits [23]. Just one review referred to culturally appropriate implementation. Liaw et al [21] reviewed 17 evaluations of the implementation of the Chronic Care Model in indigenous populations in primary care settings concluding that patients have much to benefit from the implementation of appropriate care in which cultural competence is mandatory [21]. Researchers and providers are cautioned to respect and account for cultural values and principles, the impact of colonisation (or migration) on the identity, culture and health of populations and to address individual and community-based health inequity.

In their effort to identify how constructs relate to one another and their impact on implementation outcomes, reviewers in this metanarrative began to acknowledge the multiple layers and intersections that create the complexity of implementation. In some reviews this meant explicitly acknowledging (or fully adopting) a complex adaptive systems perspective to understanding how constructs relate to one another [23]: Three reviews in this metanarrative emphasise that the context, the nature of innovations, and the capacity to sustain are interacting dynamics of a complex and unstable phenomenon [7] that is not amenable to cause- effect or command- control logic [4,24]. Suggested strategies for dealing with such complexity varied widely, including (a) the use of guidelines that are easy to use [20], (b) a sensible yet comprehensive checklist [3], and (c) acknowledgement that there may not be an optimal combination of elements in a complex care environment [19].

A final challenge represented by this meta-narrative comes in the form of a paradox: in the pursuit of establishing generalisations about factors that impact on implementation, progress toward developing useful and sensible decision tools, measures and interventions for specific, individual implementation initiatives may be lacking. The production of taxonomies of identifiable factors and ways to measure them does not suddenly make implementation easy [4], suggesting that much work remains to be done by researchers and implementers seeking to adapt this body of work to specific implementation projects.

### Metanarrative 3: Developing New frameworks from Theories, Constructs and Key Factors

The third metanarrative is based on the premise that syntheses of theories, constructs and influencing factors in the form of frameworks hold inherent utility for guiding and studying the implementation process [1,10]. The justification for reviews that sought to develop new frameworks included a common structure across publications: First, authors identify that efforts to implement best practices are insufficient or ineffective; second, authors identify that there are a wide range of previously documented elements that influence the implementation of best practices; and third, the authors suggest that a synthesis of existing literature is required to provide a comprehensive understanding of the key influential elements [25,26]. This meta-narrative includes eight publications representing 794 included studies.

This metanarrative takes its starting point from the seminal work of Greenhalgh et al [10]. Although not every paper within this metanarrative refers to that work, the wide variety of influences and disciplinary perspectives it represents are clearly diffused through this metanarrative, especially in the Consolidated Framework for Implementation Research (CFIR) [1].

The central thrust of this metanarrative is the broadening of analytic focus beyond the characteristics of the interventions and individual users to include teams, organizations, and larger environmental contexts in implementation initiatives. For example, the Interactive Systems Framework (ISF) [27] brings together broader structural and contextual features of implementation that are intended to inform a practical, consolidated process for implementing quality improvements. This broadening of focus included in reviews within this meta-narrative is reflected in a wider knowledge base including sociology [28], psychology [27], and organization and management studies [26] to further understand the impacts of context. More nuanced representations of the layers of context that influence implementation led some authors to acknowledge “the non-linear and recursive nature of the implementation process” [25]. Yet descriptions of context tended to remain simplistic and orientated toward a biomedical audience. However, this may be related to the second key dimension of this specific meta-narrative: The drive to develop frameworks that can be simply and usefully applied to practical implementation efforts.

The frameworks reviewed here sought to render the many contextual influences on implementation as understandable and usable for both practitioners and researchers by simplifying complex processes into categories to be widely applied across intervention types, health care settings, and national policy contexts [1,25]. Graphic representations of concepts as all-encompassing frameworks rendered complex relationships as understandable and usable [1]. This simplification of the influence of context represents a tension with the literature identified in meta-narrative #2, which began to address the relationships between constructs in terms of complex adaptive systems thinking. There remain divergent perspectives in the literature about the best way to deal with such complexity, with more biomedically-oriented literature selecting simplified models, and more sociologically-oriented literature acknowledging the unpredictability and complexity of contextual influences. Like in the first two metanarratives, the activities, beliefs and lifestyles of patients and families were also neglected in this meta-narrative.

### Meta-Narrative 4: Applying Existing Frameworks in Many Ways

This fourth metanarrative refers to five reviews including 143 studies of four frameworks as applied, thereby synthesising what is known about the application of frameworks into practice. The reviews included 10 studies on the Knowledge to Action (KTA) framework [29]; 24 studies on the Promoting Action on Research Implementation in Health Services (PARIHS) framework [30]; 26 studies on the Consolidated Framework for Implementation Research (CFIR) [31] and 22 studies on the use of the CFIR in implementing the Chronic Care Model [32]; and the Reach, Effectiveness, Adoption, Implementation, Maintenance (RE-AIM) framework was reviewed in 71 articles [33]. The KTA, RE-AIM and PARIHS frameworks are focused on implementation and adoption of evidence into practice, while the CFIR offers a set of constructs that are related to the full process of implementation as a means to guide the study of implementation of interventions. Four of the five studies were concerned with documenting the use of frameworks, with particular attention to whether studies adopted all domains/components of frameworks in their studies. Kadu et al [32] explore the implementation of the Chronic Care Model (CCM) using the CFIR framework.

All studies generally view theory as a means to systematize and/or improve the implementation process by providing practical guidelines or steps to be followed. All frameworks include three core domains that they suggest should be considered: 1) assessment and description of evidence/intervention; 2) context in which evidence/intervention is to be used; and, 3) the implementation process. Although each framework articulates the components of these domains differently, these domains are present across all; with the notable exception of the RE-AIM framework that does not ask for an assessment and description of evidence/intervention. The process domain of all frameworks includes “implementation” specifically and suggests that evaluation of the implementation should be addressed.

The context domain varies across the frameworks in terms of constructs with some offering a comprehensive array of contextual factors to consider (PARIHS framework and CFIR), while the KTA framework suggests general consideration of the “local context” and the RE-AIM framework focuses mainly on the number individuals adopting evidence as the main contextual factor. The reviews of the frameworks also consider context to varying degrees, but all pay attention to barriers and facilitators to implementation or adoption of evidence. The reviews exploring the use of PARIHS [30] and adoption of CCM [32] suggest that implementation is a complex process requiring consideration of how implementation fits within specific environments, considering the individuals, teams, organizational setting and the health system in which the intervention is embedded.

There is a notable tension in these articles around the dominant narrative compared to the review methods used. In the background and discussion sections of these reviews authors often highlight the importance of considering context when adopting these models. Reviews of the PARHIS framework [30] and CFIR [1,32] indeed suggest that the strength of these models is their attention to the interplay of contextual factors, which may suggest an interpretivist approach. However, all studies adopted positivist approaches with regard to the types of questions asked and the methods used to extract data and derive conclusions.

Additionally, four of the reviews were primarily intended to document the use of the frameworks in the literature without offering interpretive or critical assessments of their adoption or of the tools themselves [29,30,31,32]. This tension is particularly evident in the study reviewing the PARIHS framework [30] in which authors put forward a non-positivist view of implementation frameworks (the need for contextualized and nuanced understanding of implementation), however suggest there is a requirement for positivist studies to provide evidence of the value of the framework. Similarly Kadu et al [32] highlight facilitators and barriers to implementation of CCM at multiple levels (micro, meso, macro) using the CFIR framework, however do not explore the interplay or interpretation of these factors.

As with the previous three meta-narratives, the considerations of patients and families were not apparent within this meta-narrative.

### Metanarrative 5: Evaluating Effectiveness of Interventions within Frameworks/Models

The final meta-narrative was about evaluating the effectiveness of implementation interventions across a variety of criteria, most of which examined either (a) the extent of practice change, (b) outcomes for patients, (c) efficiency/cost related outcomes, or a combination thereof. The content of the implementation initiatives that were evaluated varied widely (as expected), with early studies consisting primarily of individual “one-off” implementation initiatives (such as introducing best practice guidelines), and later interventions being more multi-faceted and situated in comprehensive frameworks or models. The overarching message of this narrative relates to the paramount importance placed on demonstrating “what works” for the implementation of best practices. Despite the variety of approaches to supporting the implementation process, and the variety of theoretical frameworks or models informing these initiatives, a consistent effort to determine effectiveness was consistent across reviews included.

This meta-narrative included nine reviews for a total of 574 empirical studies. Articles included in this meta-narrative explored effectiveness through the use of quality improvement processes, computerized decision support, and opinion leaders as important in the implementation process being effective [34,35,36,37]. There was a limited number of studies that looked at cost effectiveness in the interventions. For those that did consider cost, there was evidence of integrated care systems providing cost savings [34,38].

Two notable themes arose in the articles included in this final meta-narrative. The first relates to the effectiveness of “single versus multi-faceted” strategies to promote implementation [33,34]. This refers to the provision of multi-component interventions to help health care providers understand new changes to practice and alter their work routines accordingly, or to simpler single-component approaches to achieving the same goals. Reviews that explicitly compared strategies grouped into these two categories (single and multi-faceted) generally reported little difference in effectiveness between the two [33,34,35], despite acknowledging the likely benefit of multi-component interventions that are informed by implementation theories. This meta-narrative included the recognition that further work was needed to determine the effectiveness of interventions that are more theoretically informed. For example, Hakkennes and Dodd [35] explained that further research “using emerging theoretical frameworks for understanding professional and organisational behaviour change may assist in determining which strategies are more likely to be effective under different circumstances” (p. 298).

The second feature of this meta-narrative related to the emerging recognition of the importance of context in determining the effectiveness of implementation interventions. Although articles falling within this meta-narrative did acknowledge the importance of, for example, tailoring interventions to the “determinants” of practice with a given setting [35], there were widely different descriptions of what counts as “context”. This point resonates strongly with the emerging attention to context in meta-narrative 2; however, in this case, context was being conceptualized as a consideration that explicitly moderates the effectiveness of implementation interventions. Authors called for further attention to context, including the ways in which it is conceptualized and operationalized within the bounds of a given implementation study.

Across the literature informing this metanarrative there are a number of limitations impacting on understanding the question of effectiveness. Firstly, there is a far greater number of studies with the aim of understanding the characteristics of an intervention that make it effective as distinct from understanding how to implement it effectively. Secondly, the vast range of approaches used to study the question of effectiveness creates challenges for comparing across studies to determine their comparative benefit. Finally, studies looking at effectiveness are more often focused on process measures as distinct from outcome measures. Particularly lacking in the reviews are papers that look at cost based outcomes, and as with the other meta-narratives, patient-specific outcomes as well.

## Synthesis and Discussion

The metanarratives above have drawn together included reviews into storylines that shed light on what can be known from systematic reviews of studies relevant to implementation frameworks, theories and models. Now we consider their higher order intersections, differences and commonalities as four themes synthesised in a way that lends further understanding about the attributes to consider when embarking on implementation. The first and second themes synthesised three metanarratives; the third theme captured all four metanarratives; and the final theme included two.

### Purpose and scope

Of the reviews included, a fundamental purpose was to build utility into the implementation process by improving components of existing frameworks or developing new frameworks [13,14]. Previous efforts to implement best practices were found to be insufficient or ineffective and there was recognition of the need to identify the components that most influence effective implementation. This led to looking beyond beyond the characteristics of interventions and individual users to include the teams, organizations, and the environmental contexts of implementation.

Existing frameworks identified general evidence, evidence of implementation context, and evidence of implementation process. Meeting the purpose was clearly shaped by the nature and complexity of the context. New frameworks attempted to represent this complexity and to be broadly applicable and portable across a wide range of intervention types, health care settings, and policy contexts. “Usability” was a pragmatic central purpose and reviews consistently sought to provide practical guidelines or steps.

Effectiveness of specific interventions, and of the implementation process, are both clear purposes in a number of reviews. Cost-effectiveness was related to the effectiveness and feasibility of an intervention, but was identified in only a small number of reviews and yet is a key consideration in the nature of primary health care that may be afforded and delivered.

A strength of this review is the confirmation that a synthesis of perspectives from a range of disciplines is essential to gain a comprehensive understanding of how policy, environmental, cultural and social contexts impact on implementation. Therefore, in developing an implementation guide for the ICOACH programme we are alerted to the need for careful assessment of the context of implementation from multiple disciplinary perspectives in order to fully appreciate the barriers and enablers that may impact on integrated primary health care for the older adult population with complex health needs. For example, the understanding that is required of the communities in which people reside is necessarily very broad and incorporates multiple factors that impinge on how health and social care can most appropriately be delivered. An understanding of what works is just one step in considering how to make an approach care actually work.

### Theory and mechanisms

The strengths of theoretically-based implementation research and practice were considered to improve health outcomes and drive advances in the science of implementation [1,2,3,4,5]. However, the evidence for how to select from the broad range of general and specific theories to inform implementation was not presented. Clear implications for policy, research and practice are that a single theory of implementation, applicable anytime and anywhere, is not feasible because of the wide variation of influences on implementation, disciplinary orientations, intended outcomes and community-based settings.

The context of implementation strongly influences how a theory may be applied within a particular project or programme [5]. An overly specific theory would have limited applicability. In spite of this, reviews of some frameworks, for example the PARIHS framework [16] and the CFIR [14,18], claimed that by accounting for the interplay of contextual factors, their constructs could be adapted when applied. Paradoxically, while a contextualized and nuanced understanding of implementation is enabled by such frameworks [16], reviews called for a high degree of control over influencing factors when testing their effectiveness. Tilley and Pawson’s approach, realist evaluation, offers an alternative whereby context is specifically addressed in the assessment of program outcomes [39].

Important findings are that while some frameworks have utility for our implementation purposes, the theory bases of existing frameworks do not comprehensively address what is needed to guide the ICOACH programme. In particular, there are gaps concerning person-centred care [40], relationship-centred care [41], and culturally safe care [42]. Other gaps may become apparent through discussion within the team and with other advisors including healthcare consumers, as we develop the specifics of our implementation framework.

### Context, complexity and process

Overall, there was strong agreement that the context of implementation is shaped by multiple influencing factors that fundamentally affect, and are affected by, the change processes used and outcomes achieved. A key concern was that patients receive appropriate care and avoid harm through identification and management of the factors that impact on the execution of innovation. The context of implementation featured as a complex adaptive system typifying the multiple interactions and patterns of healthcare in which different determinants are known to interact in ways that deny confident prediction of outcomes [8].

Alternative taxonomies for framing the context of implementation offer that the context occurs at various levels: patients and families, healthcare providers, organisations and policy-makers. Others suggest the levels of structure, organization, provider, patient, and innovation. Even healthcare consumers earned a brief mention, respect and account for patients’, families’ cultural values and principles were seldom considered, with just one review advocating for such inclusion [11].

Management of implementation as a way of controlling its complexity involves effective planning, project management and inclusion of key stakeholders. Policy-makers, implementation scientists and practitioners are urged to pay attention to careful adaptation to local conditions when interventions were not working as originally designed. Importance was placed on social and environmental interaction that favoured knowledge exchange, rather than transmission of information. Multiple parties engaging in participatory relationships seemed the key to designing and tailoring an intervention to the unique setting of patients and health professionals.

While it was disappointing that there was nothing novel or unexpected that we uncovered about the nature of context, complexity and process in this review, what we can offer is that implementation is necessarily influenced by and should be planned, well-informed, advised and actioned from multi-disciplinary perspectives by multi-disciplinary teams. What we can take from this for the ICOACH project is to continue to include decision-makers, academics, clinicians from multiple professions, providers, social services and cultural leaders and to even more strongly involve health care consumers in all phases of planning and delivery of the implementation phase of our project.

### Outcomes and Success

The included reviews identified many studies of the characteristics of interventions that made them effective, with far fewer investigating how to implement interventions. Studies of effectiveness were dominated by process rather than outcome measures and fewer still measured costs. Reviews agreed that there was small to moderate evidence for the effectiveness of some elements of the CCM, with decision support and reminder systems having the biggest consistent impact of any individual factors across the reviews. Context seemed overwhelmingly important in determining the effectiveness of interventions. In particular, health professional knowledge, motivation, and perceived benefits were considered to be key features in designing an intervention [36,43].

The development of instruments for measuring outcomes is seen as a priority [4,23] and has clear implications for implementation policy, research and practice. One suggested framework for outcome categories included [23] adoption, fidelity, cost, penetration and sustainability. There was no clear-cut set of indicators or outcome measures that categorically indicates successful implementation and neither would a ‘one-size fits all’ approach to determining success be feasible, appropriate or expected. Success of implementation does need to be shown, however, to justify funding, effort and, not least, to improve people’s quality of life.

Knowing what works and to whom it matters is a fundamental tenet of the ICOACH programme. Thus, the implementation phase of the ICOACH programme has great potential for extracting some important principles for implementing integrated care in the context of our programme of research. We can take heed of what we were not able to glean from this metanarrative review by instigating careful evaluation from multiple perspectives of our implementation processes and outcomes throughout.

## Limitations

While our search strategies were extensive we may not have located every published review on this topic. However, we found there was well-developed exposure of concepts, perspectives and premises to provide a coherent and meaningful account of implementation, consistent with metanarrative methods [8,9,10,44]. Many of the reviews included heterogeneous methods so that it was not possible to make a ‘clean’ categorisation of paradigmatic traditions within any metanarrative. However, we considered these limitations to be consistent with the ‘messiness’ [9] of metanarrative reviews.

## Conclusion

We emphasise that the strength of this article lies in drawing together multi-paradigmatic and multi-disciplinary literature in a way not done previously. The clear messages that we take from this metanarrative review to inform the iCOACH programme are that, in spite of finding no new revelations, the scope and complexity of implementation mandates robust consideration of what works, for whom, and under what circumstances, and that no existing implementation frameworks are fully comprehensive for our purposes. Further, the literature is clear that active partnership with healthcare consumers, providers, organisations and policy-makers is required. We were concerned that consumers and their families are still rarely mentioned in the literature and strongly urge implementation scientists to reverse this oversight as we are determined to. All phases of implementation of a new model of care call for collaborative adaptation with stakeholders, the most important being the person receiving care in terms of what matters most to them.

## Reviewers

Viktoria Stein, Senior Fellow Integrated Care, Head of the Integrated Care Academy©, IFIC, UK.

One anonymous reviewer.
